# NIH Resilience Model: Longitudinal Effects of Surgery on Genetics, Physical Capacity & Chronic Conditions

**DOI:** 10.1093/geroni/igaf122.3980

**Published:** 2025-12-31

**Authors:** Kenneth Seldeen, Frances Yang

**Affiliations:** University of Kansas Medical Center, Kansas City, Kansas, United States; University of Kansas, University of Kansas Medical Center, Kansas, United States

## Abstract

The National Institutes of Health (NIH) Resilience Research Working Group has put forth the resilience model that over time, a system’s dynamic response to a challenge depends on the severity of the stressor, the length of time exposed, and innate biological factors. Measuring resilience therefore creates opportunities to evaluate predictive potential for future health outcomes, relationship to underlying biology, and capacity to be enhanced through intervention. This study hypothesized that older adults (aged 65+), who are resilient will have higher physical capacity and fewer chronic health conditions over time (response) after experiencing one or more surgeries within a 12-month period (challenge) due to a better polygenics score (system). Therefore, this study examined resilience in the 2016 through 2020 waves of the National Health and Aging Trends Study (NHATS), which consists of a nationally representative sample of older adults. There were 2,827 older adults with polygenics score for BMI. Using latent variable modeling, we found that with each increase in the number of hip, knee, heart, cataract, or back surgeries during 2017 there was and associated decline in physical capacity (walking 3 or more blocks, b=-0.14, p<.001) and polygenic BMI scores (b=-0.002, p<.001 - with a response of increasing physical capacity from 2016 to 2019 with overall declines in the number of chronic conditions in 2020. These data provide new insights into the predictive potential of resilience measurement and support the goal of NIH to establish fundamental knowledge about resilience to enhance health, lengthen life, and reduce illness and disability.

